# Does artificial intelligence kill employment growth: the missing link of corporate AI posture

**DOI:** 10.3389/frai.2023.1239466

**Published:** 2023-11-17

**Authors:** Jacques Bughin

**Affiliations:** iCite, Solvay Brussels School of Economics and Management, Université libre de Bruxelles, Brussels, Belgium

**Keywords:** artificial intelligence, derived labor demand, product market competition, probit regression, contract curve

## Abstract

**Introduction:**

An intense debate has been on-going about how artificial intelligence (AI) technology investments have an impact on employment. The debate has often focused on the potential of AI for human task automation, omitting the strategic incentive for firms to cooperate with their workers as to exploit AI technologies for the most relevant benefit of new product and service innovation.

**Method:**

We calibrate an empirical probit regression model of how changes in employment relate to AI diffusion, based on formalizing a game-theoretical model of a firm exploiting the twin role of AI innovation and AI automation for both absolute and competitive advantage.

**Results:**

The theoretical game-theory prediction is that employment following AI technology adoption is not negative, and ultimately depends on how AI leads to new success in innovation, competition which defines the competitive reward of innovation and profit sharing between workers and firms. Our estimation, is based on a global survey of 3,000 large companies across 10 countries, demonstrates that a firm employment growth depends on two strategic postures, that is, the firm relative maturity of AI adoption as well as its relative bias toward AI-based product innovation.

**Discussion:**

The contribution of this research is to highlight the twin role of firm and workers in shaping how technology will affect employment. AI in particular marries the potential of task automation with even more potential for expansion.

## 1 Introduction

From John Stuart Mill to David Ricardo, economists have long been suspicious about the link between technology and jobs. Keynes (1931) then coined the term “technological unemployment” in his essay entitled *Economic Possibilities of our Grandchildren*, in which he predicted that the “most pressing problem (…) would be how to fill our leisure time” thanks to labor-saving automation. Keynes' prediction has not come through, but fears have returned with the development of artificial intelligence (AI) technologies. As in Keynes' days, the focus has remained on how AI may outcompete labor (Frey and Osborne, 2013; Brynjolfsson and McAfee, 2014; Acemoglu and Restrepo, 2018).

Recent empirical evidence confirms the limited pressure of AI on employment (Georgieff and Hyee, 2022). But part of those results may be due to the fact that AI is a General Purpose Technology (Crafts, 2021) whereby AI diffusion is costly and its effect takes time to manifest itself. What's more, even if AI does compete with labor, its current state of development makes it impossible to compete with all kinds of tasks (Bughin and Seong, 2018 or Nedelkoska and Quintini, 2018). Finally, labor can also be augmented by AI (OECD, 2019), particularly when it is used in combination with high-skill occupations (Bughin et al., 2018; Pissarides and Bughin, 2018; Balsmeier and Woerter, 2019).

Here, we hypothesize that there are some relevant employment opportunities linked to AI because AI technologies are unique in offering benefits beyond automation efficiency. Examples of AI breakthrough innovations include the rise of autonomous cars, ChatGPT, and the fast discovery of pandemic vaccines. In fact, empirical studies on previous technologies have already shown that firms exploiting technologies to launch new winning product innovations boost employment.^1^ More recently, Babina et al. (2020) seminal study on AI has concluded that AI-based product innovation correlates positively with employment growth.

Our contribution goes beyond that of Babina et al. (2020) in several ways. First, our premise is that studies should resist selecting a narrow scope, e.g. either automation alone or innovation alone, as the source of AI. Rather, we look at how corporations allocate resources between *both* benefits of AI. Second, our empirical analysis of how AI deployment affects employment dynamics is explicitly rooted in a game-theoretical framework of firm strategic investment in either form of AI. While other conceptual frameworks exist, our theory allows us to emphasize firm competition and AI investment mix, as key drivers of how AI adoption would ultimately affect corporate employment.^2^ In particular, we extend the task-based model of Acemoglu and Restrepo (2020) to account for AI-based innovation gains, and for oligopoly competition between firms. Third, our empirical test is based on a comprehensive survey of more than 3,000 large firms across multiple industries and countries. The advantage of relying on a survey is that the survey can collect data that would otherwise be not accessible, as being private information, such as for example, the degree of exploitation of each AI technology, as well as firm strategic orientation underlying its AI investments.

Other survey-based research on AI such as Lee et al. (2022), Rammer et al. (2022), or Czarnitzki et al. (2023) also collect information on the variety of AI technologies adopted. Our survey is richer as it exploits the type of strategic intent (innovation or efficiency) of AI adoption, and looks at various types of assets that could be affected by AI (e.g., capital, labor, or intermediary goods).^3^ Other studies between AI and jobs level, such as Babina et al. (2020), Damioli et al. (2022), Fossen and Sorgner (2022), and Georgieff and Hyee (2022) take a different path and assess the development of jobs based on their possible AI exposure, with the hypotheses that more AI exposed jobs should grow less than other job types if AI is especially substituting for human tasks. However, this strategy does not control for the fact that AI exposure can be linked to different forms of human skills, and does not control where exposure has materialized among corporations. Another set of studies uses statistics linked to AI patents, but this misses the much larger population of corporations using AI technologies.

The research reads as follows. We first define the methodology used. We in particular introduce the survey but also the game-theoretical model of employment change with AI adoption, which forms the basis of our empirical model for estimating how AI adoption interacts with employment. The empirical results are then presented. The final section concludes by stressing the implications of the results. We conclude by highlighting the limitations and possible extensions of this research.

## 2 Methodology primer

### 2.1 Scope

We focus on the evolution of employment due to AI. Obviously, there is more to the “Future of Work” than t the number of jobs. AI at large has supported new models of work organizations such as the gig economy (Walker et al., 2021); AI itself can help functions such as Human Resources (Eubanks, 2022). Also, the “Future of work” linked to AI must consider jobs quality (Liu, 2023), and impact on wages (Fossen et al., 2022), as the necessarily complementarity human skills to boost corporate performance (Xie et al., 2021; Babina et al., 2022; Georgieff and Hyee, 2022). Here we keep the focus on the issue of employment changes because it remains a key social concern (Lozano et al., 2021). The recent development of generative AI has made this concern more acute, with jobs originally thought to be immune like creative jobs being also at risk of substitution by AI, albeit they (Bankins et al., 2023; Bughin, 2023 or Felten et al., 2023).

### 2.2 Artificial intelligence definition

Our definition of AI is aligned with that of the OECD experts panel (2019) and stands for a set of technologies that are both technically capable of *mimicking human cognitive functions, and that would be economically attractive for companies to consider adopting*. We zoom in on five *economically attractive AI technologies for* companies to consider investing in. Those five AI technologies are the common list among the various academic surveys on AI adoption, such as Lee et al. (2022), Rammer et al. (2022), and Ameye et al. (2023). The AI technologies are part of the taxonomy used by major official statistics institutions, worldwide.^4^ The first is software-based robotic process automation (RPA). RPA is often associated with pure automation, as the software is often directly integrated into physical robots. The second type of technology includes chatbots or virtual assistants; the third concerns computer vision, while the fourth is natural language recognition and processing which recently got a boost as generative AI, through new encoders. Last but not least, the recent advances in AI are mostly based on neural network-based machine-learning techniques.

### 2.3 Two steps approach

Our study relies on two unique pillars of theory and empirical verification of how profit-maximizing AI adoption and postures affect the link between employment and AI, as described below.

#### 2.3.1 Theoretical foundation

Based on the literature review, we have chosen to develop a game-theoretical model of AI investments that explicitly includes the dual benefits of efficiency/automation and innovations for firm competitive advantage, and how those effects shape the “derived labor demand” impact. Frequently used approaches such as the TOE framework explicitly consider competition, and organizational capabilities in the context of technology, but remain conceptual, while we seek to tie AI and employment dynamics based on *explicit firm behavior* in exploiting the benefits and risks of AI technologies.

Among other features, the model is an extension of the task-based model of Acemoglu and Restrepo (2018), but where the firms are competing among rivals, and investing in different forms of AI (automation versus innovation). In the face of rivalry, firms may strategically cooperate with workers, through revenue-sharing incentives, and sink AI resources to win exclusive innovation rights, for example. Those features are often used strategies in the context of technology, with the goal of bringing competitive advantage over rivals.

#### 2.3.2 Survey scope

The research relies on an online corporate survey that gathers detailed information about the AI technologies adopted and used, as well as the strategic direction firms anticipate by adopting AI. The survey is broad, covering 3,000 large firms from 10 countries and 18 industries. It is known that AI technology diffusion depends heavily on company size (Zolas et al., 2021), especially at the early stage of commercial diffusion. Our sample is biased toward large firms with about 40% of the sampled companies working with more than 1000 employees.^5^ As seen in Appendix 1, this study includes countries such as the US, Canada, the UK, France, Italy, Germany, and Japan. These countries are at once the largest contributors to global GDP, but also the most digitally advanced. This survey is complementary to studies at the global macroeconomic level, eg for all OECD countries in Georgieff and Hyee (2022), or single-country studies, such as the US in Babina et al. (2020), Germany in Rammer et al. (2022).

We focus on the early years of AI diffusion, as the data for this research were collected before 2017. While AI technologies have continued to improve since then, and AI diffusion is much broader by now, early studies of the effects of AI on performance a*nd* employment have the same comparable time frame (Babina et al., 2022 concentrate up to 2018 as are data in Czarnitzki et al., 2023). The survey was conducted online on a panel of 12,000 large companies worldwide by March–April 2017 by the global market research firm TNS on behalf of McKinsey and Company to explore the status of AI adoption by enterprises. This panel is stratified based on the size distribution of firms by country. The survey constructs and questions were approved by a panel of AI experts and industry specialists from McKinsey. The TNS panel is her own and ensures complete anonymity in order to limit the risk of disseminating specific confidential information. This anonymity warrants trusted survey answers. In fact, the final validated filling rate of the survey was 25.6%, or more than 3000 firms. Respondents to the questions were either from the executive board level, or, if not, from the head of technology. In this latter case, it was either the Chief Data Offfice, or Chief Technology/Digital Officer. In total, 19% of respondents were CEOs.

The agency typically carried out multiple checks based on a mixture of machine learning techniques to detect careless responses, on the basis of the speed of answer or anomaly in patterns of answers (Huang et al., 2015). We also used the common method of Podsakoff et al. (2012), in addition to a common latent factor analysis to spot any systematic response bias. The common factor found to be capturing <17% of the variance in answers, or a level that is sufficiently small to warrant survey validity.^6^

We also believe that the sample is quite representative of large global firms and how they make use of AI. Our sample reports that Chinese and US companies were the most advanced in exploiting AI, which is consistent with other research indicating that the most buoyant markets for AI investment are in these two geographies. Similarly, the most digitally mature sectors, telecommunications, high-tech, and media, were also the most advanced in using AI, in line with Kabalisa and Altmann (2021).

Table 1 gives an overview of the AI exploitation funnel, spitting between experimentation and commercial exploitation of AI. AI chatbot technology was the least known, and NLP was the most known, but the least used. This pattern of AI technology exploitation fits with other studies (Zolas et al., 2021; Mucha and Seppala, 2022).

**Table 1:** AI funnel diffusion, 2017.

**Funnel stage**	**AI Technologies**		**Language processing**	**Robotics**	**Virtual agents**
	**Machine learning**	**Computer vision**			
Not aware	29%	24%	5%	12%	42%
Not yet invested	29%	39%	43%	12%	29%
Pilot	15%	14%	18%	14%	11%
Adopted for a single use case	12%	12%	18%	28%	10%
Adoption of many use cases	15%	11%	16%	34%	8%

Table 2 shows the industry picture of how companies anticipate employment to change as a result of their AI adoption in the next 3 years. The largest portion of respondents believe that AI adoption *will not* impact *total* employment in their firm while for those anticipating changes, the effect seems to be a slight net negative. Other surveys of employers carried out at the same time as this research show more or less the same pattern of expected employment changes due to AI, e.g., a ManpowerGroup (2017) study worldwide has found that about 50% of companies will see no change in the next two years due to AI, and around the same portion will see a decline, or increase. In another survey conducted by ServiceNow (2017),^7^ about 1/3 of companies stated that they will change their level of employment as a result of AI diffusion.

**Table 2:** Expected employment direction as result of AI.

	**Effects on employment (Next 3 years), % of responses**			**Higher**
	**Lower**	**Same job**	**No effect**	
Sectors	Employment	but some areas are changing		Job
Construction	25	24	40	11
Media	24	25	43	8
Telecommunications	24	32	25	19
Automotive and	23	34	33	10
Transport	22	26	38	14
Education	20	20	54	6
High technology	19	32	32	17
CPG	19	33	36	12
Energy	19	33	41	7
Financial services	18	32	41	9
Travel	17	30	41	12
Retail sales	16	26	49	9
Health	14	28	53	5
Professional services	13	14	66	7

Source: Survey, author's calculation.

Finally, the survey asked respondents to list all strategic rationales for adopting AI (Table 3). In all responses, *market share expansion is as important a motive as workforce efficiency* when it comes to deploying AI. The importance of innovative opportunities beyond efficiency is thus justifiable as a strong motive to adopt AI, and as supported elsewhere, such as in Bag et al. (2021) and Kabalisa and Altmann (2021).

**Table 3:** Strategic intent of AI adoption, 2017.

**Sector**	**Revenue growth (%)**		**Efficiency gains (%.)**		**Raw materials**
	**Market share**	**Market size**	**Capital**	**Labor**	
Telecom	23^*^	28	20	20	8
Media	17	29^*^	14	17	22
Financial Services	23^*^	23	16	30	9
CPG	26^*^	19	19	26	10
Retail sales	20	20	24	20	16
Professional Services	21	16	21	21	20
Education	17	20	27	16	20
High technology	14	21	27	24	14
Health	20	15	24	32^*^	9
Travel	10	24^*^	17	21	27^*^
Energy	21	12	24	24	18
Transport/logistics	24^*^	7	24	30	15
Automotive	16	12	25	21	26
Construction	8	8	30^*^	27	27^*^

*/° : statistically superior/inferior to the industry average.

## 3 Theoretical foundations

The survey overview is consistent with the idea that firm employment is shaped by firms' strategic use of AI. In this section, we formalize this intuition by laying out a simple model of derived labor demand for a firm competing in an oligopoly. The model extends traditional derived demand models (Van Reenen, 1997; Ugur et al., 2016), to AI technologies, adding endogenous production technology whereby tasks are allocated according to the relative productivity merits of AI automation/innovation mix (Zeira, 1998; Acemoglu and Restrepo, 2018; Babina et al., 2020).

### 3.1 Hypotheses and notations

We consider the i-th firm, maximizing its profit Π_i_, and competing with n−1 other firms. The firm produces a quantity, Q_i_, as a contribution to a market output of Q, with a market demand exhibiting a price-elasticity κ, (1/κ < −1). The product is homogenous among firms, but firms will invest in better quality products through the leverage effect of AI.

The company's product is made through a combination of micro-tasks. Those micro-tasks, ranked from 0 to 1, are supplied either by labor, *L*_i_, at unit cost, w_i_, or by AI, at rental cost r. We note 0 < σ = 1 as the elasticity of substitution among tasks, and thus between types of inputs^8^. The level and mix of AI investment is chosen by the firm before the firm competes on the product market. Firms can only adjust employment in response to product market dynamics.

Given sunk investment costs, firms play a Nash quantity game (Maskin and Tirole, 1988 or Herk, 1993). Since labor is fully variable, the final wage determines the firm's marginal cost. As recognized in the economic literature, firms therefore have an incentive to design a wage structure with a base plus a profit-sharing system, 0 < ϕ < 1 negotiated with employees. This lower base acts as an optimal signal that the firm wishes to compete more aggressively against rivals (Bughin, 1999; Ferreira and Waddle, 2010). Empirically also, this fits with the practices of many technology firms to support their growth (Aral et al., 2012; Wang, 2016). In the particular case of AI automation, higher labor productivity achieved per unit of labor also hedges against the risk of automation, for 0 < ϕ, and thus is also compatible with workers' preferences, if any, for employment.

If the firm does not adopt AI, the firm only uses labor, and we note the average productivity of labor by ρ > 0. When adopting AI, the firm decides on the level and mix between AI for efficient automation (AIE) and for innovation (AII). While AIE is essentially invested to reduce unit cost by automation and the use of automation is accessible to any firm, AII returns depend on winning the innovation race in order to secure exclusive innovation returns. As in models of vertical differentiation (e.g., Wauthy, 1996), exclusive innovation win acts as a shift in sold output, and thus boosts firm market share against rivals from 1/n to (1 + vγ)/_n_ where γ= n – 1 > 1 is the extent of innovation quality^9^ and 0 < v < 1 is the probability of successful exclusive innovation.

Finally, as in Zeira (1998) and Acemoglu and Restrepo (2018), let us assume that tasks are organized such that higher tasks in the space (0,1) improve the comparative advantage of labor, while it decreases the value of AI innovation. Aggregating over all tasks, a Cobb Douglas-like production emerges:


(1) $$\begin{array}{c} Q_{\text{i}}=A.{\left(1{/}_{\left(1-\alpha \right)}.L_{\text{i}}\right)}^{1-\alpha }.{\left(1{/}_{\left(\text{αβ}\right)}AII_{\text{i}}\right)}^{\text{αβ}}.(1{/}_{\left(1-\beta \right).\alpha }AIE_{\text{i}}^{\left(1-\beta \right).\alpha } \end{array}$$

where A is a drift representing the share-weighted product of input productivities. The share 1–α, (0 < α < 1) is produced by labor, L, and thus corresponds to tasks with sufficient high comparative human advantage. The share β is determined by when expected gain of innovation is such that vγ > ψ and ψ is the average productivity of AIE.

### 3.2 Equilibrium

Given the sequence of the game, the model must be solved backwards in order to ensure a subgame-perfect equilibrium. Thus, we first solved the last stage, when firms determine output and wages, while the early stage of AI investment is discussed thereafter.

#### 3.2.1 Employment and wages

Remember that wages are linked to profit sharing. As shown for example in Gottfries and Sjöström (1995), the optimal real wage, w_i_, is equivalent to a wage resulting from a generalized Nash bargaining contract curve between the firm and the workers, and 1 ≥ϕ≥ 0 is the profit sharing share. In the absence of unemployment benefit, the alternative wage, aw_i_, is to be seen as wages times probability of employment. Thus, given (2) above:


(2) $$\begin{array}{c} aw_{\text{i}}=w_{\text{i}}.\alpha \end{array}$$

The wage and contract curve solutions are the first-order conditions set to zero of the Nash bargaining solution:


(3) $$\begin{array}{c} {\left[\left(1-\alpha \right)w_{\text{i}}L_{\text{i}}\right]}^{\text{ϕ}}\Pi_{\text{i}}^{1-\phi } \end{array}$$

Using (2) – (3), the usual results emerge that the labor share is linked to the bargaining power, ^ϕ^ (McDonald and Solow, 1981):


(4) $$\begin{array}{c} w_{\text{i}}L_{\text{i}/\text{PQi}}{=}^{\text{ϕ}}\left(n-\kappa \right){/}_{\text{n}} \end{array}$$

and the Nash equilibrium contract curve output, Q_i_, meets:


(5) $$\begin{array}{c} P.\left(n-\kappa \right)=n.\alpha .w_{\text{i}} \end{array}$$

Equation (6) is the traditional markup formula under oligopoly but where marginal cost is the alternative wage, α.w_i_, and α.w_i_ < w_i,as_α < 1. Furthermore, using (2):


(6) $$\begin{array}{c} w_{\text{i}}L_{\text{i}}/Q_{\text{i}}=\left(1-\alpha \right){/}_{\text{α}}\; \end{array}$$

(7) $$\begin{array}{c} w_{\text{i}}L_{\text{i}}/PQ_{\text{i}}=\left(1-\alpha \right).\left(n-\kappa \right){/}_{\text{n}} \end{array}$$

and it follows from equalizing (5) and (6b) that:


(8) $$\begin{array}{c} \alpha =1-\phi \end{array}$$

Thus from (7), δα/dϕ < 0, or workers bargaining power pushes for more employment. Said otherwise, the cooperative agreement between workers and the firm on the labor contract curve secures slightly more employment in exchange for a lower wage base rate, which makes the firm signal lower costs and thus more aggressive and profitable output sold.

#### 3.2.2 AI investments

As discussed, AI investment types have different appropriation rates. Process automation can be done by any firm, but firms may protect their innovations for a unique competitive advantage. We follow Delbono and Denicolo (1991) in postulating that technological investment should also increase the chances of success, v, of innovation by accelerating innovation throughput. For any θ measuring the time effectiveness of the innovation spent, we note:


(9) $$\begin{array}{c} 0<v_{\text{i}}=1{/}_{\text{θ}}{\left(AII_{\text{i}}\right)}^{\text{θ}}<1\; \end{array}$$

From (7), dv/dAII < 0; so that there is a maximum to spend on AII. The optimum is achieved when dEΠ_i_/dv_i_= r. AII_i_, and where the expected gross profit, EΠ_i_ is estimated at optimum, that is EΠ_i_ = P^*^.Q^*^.k.(1+vγ)/*n*.

This leads to:


(10) $$\begin{array}{c} PQ.k.\gamma /n{\left(AII_{\text{i}}\right)}^{\text{θ}}=r.AII \end{array}$$

Integrating back into (8), the firm chooses v such that:


(11) $$\begin{array}{c} PQ.k.\gamma /nv\theta =r.AII \end{array}$$

Given the Cobb Douglas (2), we also know that for each type of AI capital,


(12) $$\begin{array}{c} AIE_{\text{i}}=\left(1-\beta \right).\alpha Q_{\text{i}/\text{r}}\; \end{array}$$

(13) $$\begin{array}{c} AII_{\text{i}}=\beta .\alpha Q_{\text{i}/\text{r}} \end{array}$$

Thus, integrating (9b) into (11),


(14) $$\begin{array}{c} \theta .v=\beta \alpha n/Pk.\gamma \end{array}$$

Using (7), we find:


(15) $$\begin{array}{c} \beta =\theta .v_{.}Pk.\left(n-1_{\text{n}}\right)/\left(1-\phi \right). \end{array}$$

And the allocation of AI spent toward AII relative to AIE depends positively on the throughput ability θ, the success of innovation, v. P.k.(n–1/n), as well as on profit sharing, ϕ > 0. Thus profit sharing biases AI toward AI innovation.^10^

#### 3.2.3 Integrated solution

The complete equilibrium is found by reinjecting the optimal allocation parameters in (7)–(12) into (4) and (2), so that we can in particular focus on how employment is then linked to AI spent in equilibrium. Starting from a situation of no AI, (2) tells us that AI leads to a reduction in the proportion of α, which evidently reduces employment for the *same* level of output. But, the real trick is how AI leads to output expansion that will further boost employment as a new level of average labor productivity.

Let us note: η= (n – 1)/n (1 – ϕ)), which is a measure of shared innovation gains between workers and the firm. Normalizing *P* = 1 for readability, transforming (2) in log form, and using (7), (12) which defines the endogenous parameters of the task-based Cobb-Douglas production function, the effect on employment is positive when:


(16) $$\begin{array}{c} \left(1-\phi \right).\ln\left(\psi /\rho \phi \right)+k\eta .\theta .v.\ln\left(v\left(n-1\right)\psi \right)>0 \end{array}$$

Rearranging:


(17) $$\begin{array}{c} \left\{1-\phi \left(1+k_{.}\eta v\right).ln\left(\psi \right)k_{.}\eta \theta .v_{.}ln\left[v\left(n-1\right)\right]\right\}.ln\left(\rho \phi \right) \end{array}$$

Under the assumptions of perfect competition and only AI automation, as in Acemoglu and Restrepo (2018), the expression (13b) collapses to:


(18) $$\begin{array}{c} \psi /\rho >\phi =\left(1-\alpha \right) \end{array}$$

Making clear that the effect of AI on employment *turns out to be positive* if the ratio of AI automation to labor productivity is sufficiently large, versus employment reduction following AI automation adoption.

When one relaxes the restrictive assumptions in Acemoglu and Restrepo (2018), such as in (13b), employment expansion following AI extends to a further number of critical channels, such as:

1. Innovation success, (υ) which itself depends on both the level and effectiveness of AI spent toward innovation,
2. Competition (τ, n), which defines the competitive reward of innovation
3. Profits sharing (ϕ), which synthesizes how firms and workers share benefits of AI including lower risk of AI automation substituting labor
4. Product demand elasticity (κ), in that a higher price elasticity leads to more production and employment

The above theoretical insights should form the backbone for our empirical analysis of how employment is likely to evolve in line with relative AI adoption/investments by firms.

## 4 Empirical results

### 4.1 Empirical specification

Define I as a categorical variable coded as “1” if the change in corporate employment is positive, “0” if no effect, and “-1” if negative following AI adoption. Then, based on the theory, we can derive a model in the ith firm such that:


(19) $$\begin{array}{c} I=a_{0}+a_{1}COVERAGE+a_{2}.DIFFUSION_{\text{i}}\\ +a_{4}MARKETEXTENSION_{\text{i}}+a_{5}MARKETSHARE\\ +a_{6}.NLEFFICIENCY_{\text{i}}\\ +a_{7}.LEFFICIENCY+a_{8}MARKUP+a_{9}CONTROLS+u \end{array}$$

Where u is an error term, and a's are coefficients to be estimated. As for the right-hand-side variables, and on the basis of our survey, we first include two (extensive and intensive) measures of the extent of AI adoption. The first, COVERAGE, lies between (0,1), and =0 if firms have adopted none of the AI technologies, and reaches 100% if the company has adopted the five AI technologies covered in our survey^11^. The second, DIFFUSION is 0, when the adopting firm is only exploring/testing any AI technology and equals 1 if the adopting firm is exploiting all the five AI technologies for commercial use cases.

Our survey does not measure AI success. Rather, we exploit the set of objectives set by firms when investing in AI. Thus, AI innovation is measured by two binary variables: MARKETEXTENSION which equals one if firms are using AI to expand the product market space, and MARKETSHARE which equals one if the goal is to gain market share against competition. Regarding the use of AI for efficiency, we build the dummy variable LEFFICENCY = 1 if the firm uses AI for labor automation. We also capture whether firms would use AI for other inputs' efficiency gains, in capital assets and raw materials. We compute NLEFFICIENT = 0,1,2 if AI is also leveraged for efficiency in both non-labor inputs.

We also include firms' profit margin, PROF, as a proxy for competitive intensity.

Equation (14) is estimated at the firm level as a cross-section of large firms by 2017, in line with other works studying technology adoption on employment (Barbieri et al., 2019). Other surveys based on AI and employment are also cross-sectional, (Lee et al., 2022 or Czarnitzki et al., 2023). Although cross-sectional prevents the assessment of causality and long-run dynamics, the questions in the survey explicitly clarified the causality from AI to employment. Specifically, the question raised to respondents is whether they would anticipate “job change in the next 3 years *caused by her corporation's adoption of AI technologies*”. The three-year window reflects the idea that there is a lag between technology adoption and economic impact.

Nevertheless, we also present results where we instrument AI COVERAGE AND DIFFUSION. The first instrument available through this research is the amount of AI adoption/exploitation done at the global level. The literature on technology diffusion has demonstrated a fairly strong herd behavior in technology adoption (Battisti and Stoneman, 2003 for process technology and more recently for AI, Lee et al., 2022; Ameye et al., 2023; Czarnitzki et al., 2023). The amount of AI adoption is computed, outside the industry of the focal firm. This type of instrument is referred to as the Bartik instrument, and has been used already in the AI literature (Parteka and Kordalska, 2023). The second instrument is the investment in past digital technologies that serve as complementary platforms to AI, in particular cloud, and big data given the critical importance of data for AI algorithms (Gries and Naudé, 2022). Finally, the fact that companies are faster to invest in AI if they are already more vested in previous digital technologies has also been noticed in various research e.g., Bughin (2018).

We finally include a vector C of controls to limit estimation bias risks linked to omitted variables. We control for firm headquarters location, firm size, and industry. The size variable is categorical with eight employment categories, from 0-10 employees (small firms) to very large firms (over 10,000 employees). Firm location includes 10 countries, and we use 14 NACE 2 industries. Industry is critical as it captures effects such as rivals' AI adoption, and industry level of elasticity of substitution between labor and various capital inputs.

### 4.2 Results

We use an ordered probit regression model to allow for the fact that employment outcomes are not random (see e.g., Alonso-Borrego and Collado, 2002, or Morikawa, 2016). Table 4 presents the main results, as marginal effects. We extend the baseline model results in column 1, with a model including variable interactions (column 2), as well as by instrumenting the AI adoption variables (column 3).

**Table 4:** Impact of AI on expected employment growth, probit results.

	**Base line**		**With cross-effects**		**IV plus**	**Cross effects**
COVERAGE	0.052	(2.12%)^**^	0.021	(1.07%)^*^	0.029	(0.81%)^***^
DIFFUSION	0.065	(2.78%)^***^	0.039	(1.22%)^***^	0.053	(1.14%)^***^
NLEFFICIENCY	0.001	(0.00%)^*^	0.005	(0.31%)	−0.028	(0.86%)^***^
LEFFICIENCY	−0.025	(−0.98%)^**^	−0.024	(0.08%)^***^	−0.025	(−0.11%)^**^
MARKETEXTENSION	0.030	(1.71%)	0.007	(0.18%)^***^	0.009	(0.23%)^***^
MARKETSHARE	0.049	(1.88%)^***^	0.011	(0.72%)	0.013	(0.33%)^***^
MARKUP	−0.012	(−0.56%)^*^	0.004	(0.97%)	−0.014	(−0.51%)^**^
COVERAGE/DIFFUSION	N.A.		0.004	(0.11%)^***^	0.012	(0.36%)^***^
COVERAGE/NLEFFICIENCY	N.A.		0.005	(0.44%)	0.013	(1.1%)
COVERAGE/LEFFICIENCY	N.A.		−0.024	(−1.13%)^*^	−0.028	(−0.99%)^***^
COVERAGE/MARKET EXTENSION	N.A.		0.003	(1.65%)^*^	0.015	(1.1%)
COVERAGE/MARKETSHARE	N.A.		0.049	(2.03%)^**^	0.036	(1.10%)^***^
DIFFUSION/NLEFFICIENCY	N.A.		0.006	(0.34%)^*^	0.004	(0.23%)^*^
DIFFUSION/LEFFICIENCY	N.A.		−0.019	(−0.76%)^**^	−0.015	(−0.50%)^***^
DIFFUSION/MARKET EXTENSION	N.A.		0.027	(1.44%)	0.015	(0.87%)
DIFFUSION/MARKETSHARE	N.A.		0.031	(2.41%)	0.033	(1.71%)^*^
LEFFICIENCY/NLEFFICIENCY	N.A.		−0.010	(0.74%)	0.002	(1.09%)^*^
MARKETSHARE/NLEFFICIENCY	N.A.		−0.009	(−0.65%)	−0.009	(−0.78%)
MARKET EXTENSION/NLEFFIENCY	N.A.		−0.014	(0.87%)	0.008	(0.57%)
MARKETSHARE/LEFFICIENCY	N.A.		0.017	(0.44%)^***^	0.016	(0.56%)^***^
MARKET EXTENSION/LEFFIENCY	N.A.		0.006	(0.34%)^*^	0.008	(0.61%)
MARKETSHARE/MARKETEXTENSION	N.A.		0.041	(1.26%)^***^	0.035	(1.12%)^***^
Cst	−0.151	–(0.04)^***^	−0.060	(−0.03)^*^	−0.056	(−0.02)^***^
Pseudo R^2^	0.271		0.422		0.469	
Country/industry dummies	Y		Y		Y	

Markup has no significant interaction term and removed for clarity.

Robust standard errors laid out in parentheses.

* *p* < 0.1;

** *p* < 0.05;

*** *p* < 0.01.

IV column uses the computed value of ADOPTION AND DIFFUSION as estimated in a first stage (results shown in Appendix). Test of IV validity, Hansen J's p-value is 0,672.

The pseudo-McFadden R^2^ for the three probit estimations demonstrates the importance of the cross-effects, as well as the value of the IV technique. Formally, a right tail chi square likelihood test with 15 degrees of freedom representing the cross-effects supports the more general model at the 1% threshold, as represented in either column 2 or 3. Likewise, an F-test of the joint significance of cross-effects is statistically significant at 1%. Appendix 2 lays out the first stage regression linked to IV, and strongly supports the relevance of instruments, e.g. we notice social contagion in the adoption of AI, and the foundational role of early cloud and big data technologies. For both columns 2/3, an F-test of the instruments of the focal firm AI maturity is also statistically significant at the 1% level, and largely above the typical threshold of relevance (*F* > 10). Alternatively, a Hansen J-test does not reject the instrument validity at 1%. In general, a large portion of the reported effects are significant at traditional thresholds and in line with theoretical predictions.

#### 4.2.1 Base case

For the baseline in column 1, the constant is statistically negative meaning that companies in the sample exhibit a *negative* employment trend, after correction for control effects such as industry and country effects. However, we also find that AI adoption and diffusion combined *increase* the probability of employment growth at the margin, by 12%,- or just short of offsetting the overall negative trend in employment.

In particular, the orientation of AI toward the quality of products to increase market share, boosts employment growth at the margin by 5%, so that in total, a marginal increase in AI diffusion orientated toward product innovation is enough to reverse the negative trend on employment. AI used to automate labor marginally increases the probability of negative expected employment growth, as *opposed to the* product effects of AI. Mark up effect has a limited effect, but is contrary to our theoretical model prediction.

#### 4.2.2 Extended case

Instrumental Variable (IV) exhibits the best fit, and confirms that AI adoption/diffusion is often enough to make employment growth positive. However, AI automation increases the risk of employment exhaustion versus the baseline case, while AI spent toward product innovation on employment prospects is smaller than in the baseline. The cross-effects of AI innovation with adoption and diffusion are also significantly positive on employment, suggesting that the effect of AI innovation requires some minimum adoption/diffusion to scale employment growth.

Table 5 calculates the total marginal effect of the variables, comparing results with and without cross-effects. The inclusion of cross-effects makes all efficiency factors more probable for the focal firm to have lower employment expectations, but the interactions with two variables (AI diffusion and market share gains objective) compensate for this effect in favor of employment growth. Note also that the cross-effects which contribute the most to the positive change in employment are all anchored in market share gains orientation. Finally, we also witness that the combination of market expansion and market share gains are complementary to *boost* the probability of higher expected employment growth.

**Table 5:** Marginal impact on probability of employment change.

**Variables**	**Without interaction**	**With interaction**
COVERAGE	5.19%	4.80%
DIFFUSION	6.45%	7.55%^**^
NLEFFICIENCY	0.06%	−2.94%^***^
LEFFICIENCY	−2.46%	−3.12%
MARKETEXTENSION	3.03%	1.91%^***^
MARKETSHARE	4.92%	8.05%^***^

MARKUP omitted as no significant interaction term.

Results based on Table 4.

Interaction computed as average of non IV and IV.

Statistical test based on mean difference: ^*^p < 0.1;

** *p* < 0.05;

*** *p* < 0.01.

### 4.3 Discussion

The theoretical model presented in this paper has highlighted the importance of firm strategic behavior regarding how AI would affect employment prospects. In particular, firm cooperation with workers as well as prospects of successful AI innovation, may lead to a win-win whereby firms and workers expand firm output and employment, despite the introduction of AI as labor automation. The empirical analysis has confirmed those hints. We now discuss the significance of those results:

#### 4.3.1 Results sensitivities

Using the main results in Table 4, comparative statics along the two main firm strategic dimensions (firm current AI stage of diffusion and firm AI investment mix) suggest the following:

1. AI maturity. The extent of commercial use, -rather than AI adoption-, matters the most to increase employment prospects *at the margin*. However, in the current sample, the state of commercial use is limited, at 16%, or twice lower, than AI adoption coverage, at 33%, and thus, the average estimated effect has remained minimal by 2017.
2. Since then, AI adoption and commercial diffusion have nearly tripled according to multiple business sources^12^. Using this growth impact in our estimated equation, the employment growth will happen 30% more often than in 2017, of which 2/3 of this effect would come from commercial diffusion.
3. AI-based innovation. The difference in firm probability to boost employment will be *15% higher* for a firm exploiting AI for product innovation, than for a firm not using AI. The effect is surprisingly larger for innovation associated with the current product market, than for innovation aiming at new products/new markets.
4. Automation. We also find that increasing automation reduces employment prospects, but also substitutes for other non-labor inputs, such as physical equipment. However, this can make sense: as an example, when Amazon acquired Kiva, reducing logistic handling tasks, a reduction in plant capacity also followed, with robots able to operate with 50% less space than before.

#### 4.3.2 Benchmarks comparison

The most extreme case is between a firm not adopting any AI versus another one adopting and fully using AI for commercial product orientation- the first one will have a 6% probability of job loss in the next 3 years, while the other will have an employment positive prospect increased to 16%. Between both cases, this implies a difference in better employment of 22% in the next three years.

We find that the estimated effects on employment changes are in the range of peer research, even if the comparison must be done with caution because of differences in time and methods.

Regarding the effect of pure automation, for example, studies linked to the impact of robotics on jobs have demonstrated a negative effect on employment, with an estimated magnitude of 0.3–0.5% of the active population per year in the US (e.g., Acemoglu and Restrepo, 2017). A large part of this effect is however driven by the automotive sector, and the effect of automation on employment has actually turned to be positive in recent years (Chung and Lee, 2023).^13^

Other methods based on task and occupational content (Arntz et al., 2016; Manyika et al., 2017) conclude that the employment loss when AI technology is fully exploited could be around 15%−20% of existing jobs. At expected diffusion through years, this would imply 2 percentage points of job losses per year, in line with this study, which estimates about 6% of existing job losses for the next 3 years. Empirically, Fossen and Sorgner (2022) for the US compute that digital technologies automation has increased the monthly risk of transitioning to unemployment in the US by 5%, while Yang (2022) found that AI automation in Taiwan has a very small negative effects on employment *growth*, but the effect is not statistically significant.

As far as product innovation is concerned, the literature has been cautiously optimistic about its effect on employment (Vivarelli, 2014; Herstad and Sandven, 2020), but only a few studies look at the employment effect from AI. Based on the study by Babina et al. (2020), the portion of jobs associated with AI product innovation has grown by 3% a year. Our estimates are somewhat higher than those by Babina et al. (2020), but their estimates are based on much earlier data from 2010-2018. Looking at studies of AI patents, as a proxy for AI innovations at large, one observes a boost in jobs in the range of 4–8% a year (Damioli et al., 2022; Yang, 2022).

#### 4.3.3 Strategic and managerial implications

The strategic and managerial implications at the firm level of the study are as follows. Firstly, as relative AI diffusion is correlated with employment, the study implies that firms should pace their adoption with regard to how their peers innovate with AI. This fits the argument in Bughin (2018) that, under technology-induced hyper-competition, “no AI, more than AI, is the end of job”. The recommendation is not about herding (Ameye et al., 2023), but more about the claim that the firm should watch out not to be behind competition in the adoption of AI.

The second implication is that workers and managers should consider cooperating with each other around AI. The fear of job loss is not as large as often claimed, but AI can be used for shifting jobs more toward innovations. As innovation returns may be private, e.g. through patents, the design of workers' compensations, eg in the form of either ESOP, profit sharing, or sales commission, may be of interest. Job guarantee may also be part of a new contract between firm shareholders and its workers.

## 5 Conclusions

This research tests the narrative of a “laborless future” by examining how investments in AI can affect *the demand for labor*. We have argued for taking a formal, game theory lense as technology adoption and commercialization are ultimately driven by firm interest to better compete against rivals.

The second contribution is to provide an empirical test on employment changes for a large sample of 3,000 firms worldwide, based on AI objectives, state of AI adoption, and commercial use, for five key AI technologies (machine learning, robotic automation, chatbots, NLP/NLG and computer vision).

In general, we find that firm AI posture, combined with the relative AI stage of diffusion, *are critical mediators* of how AI may shape employment dynamics. Not controlling for those effects may significantly distort the narrative of how AI affects employment dynamics, as AI innovation plays the opposite way of AI automation, for example. Furthermore, corporations might be better off launching their AI transformation earlier than later, possibly with a bias to exploit AI capability for new powerful innovations.

The research has notable caveats and scope limitations, which also suggest avenues to pursue new research. First, the sample is concerned with the early stage of commercial diffusion of AI (in this case, the year of 2017) and is cross-sectional. While the timeframe fits the early scholarly literature, AI diffusion has increased significantly in recent years, spreading as well to Small and Medium-sized enterprises. Also, AI performance may have improved significantly, possibly increasing its effect on labor dynamics. One such trend is the development of generative AI, that is changing the nature of jobs such as software coding, marketing, and customer care (Brynjolfsson et al., 2023; Bughin, 2023; Susarla et al., 2023).

Second, our data are all self-reported, and could not be matched with actual patterns of job changes. If we mitigate this risk by looking ONLY at the *magnitude* of job changes linked to AI, we were only able to provide an estimate on employment prospects, but not on the magnitude of changes, as indicated in other studies (e.g., Yang, 2022). Third, our theoretical model is at the task level, but our estimation is at the level of jobs (defined as a bundle of tasks). Task distribution is more or less linked to job skills, so the evolution of employment by type of skills may be a very valuable extension (Babina et al., 2022). Fourth, the scope of AI on the workforce is much broader than employment only. As we pointed out in our theoretical model, AI may be associated strategically with new forms of contracts. Similarly, employment can be associated with intangible elements such as experience and tacit knowledge, which could make jobs more resilient to technology in the short term (Autor, 2015).^14^ In this case, employment dynamics will be more about reallocation (Bughin et al., 2018; Pissarides and Bughin, 2018; Balsmeier and Woerter, 2019), As an early check, we have estimated a simple probit model (=0, no effect; 1 reallocation) among the sub-sample (44%) of firms reporting *no effect* of AI (see Appendix 2). The effect of AI innovation and diffusion on job reallocation is twice larger than the effects found on employment change, confirming that reallocation of work is possibly as crucial as job change.

Finally, the approach taken here is one where AI is differentiated from other types of technologies by the fact that AI marries both labor automation and augmentation. One can model AI based on its many other technical elements, such as data, architecture, and machine learning algorithms (Gries and Naudé, 2022). All this is left to future research.

## Data availability statement

The raw data supporting the conclusions of this article will be made available by the authors, without undue reservation.

## Author contributions

The author confirms being the sole contributor of this work and has approved it for publication.

## Footnotes

^1^For PC-based technologies, see Spiezia and Vivarelli (2000), Garcia et al. (2002) and Peters (2004) or for physical robots, CAD and flexible manufacturing, see Beede and Young (1998).

^2^An illustration of how competition matters in AI is the market for translation. While deep learning-based translation startups such as Deepl or Leego, have been invading the translation market in the last three years, they find themselves aggressively challenge by OpenAI using large language models.

^3^As an example why this matters empirically is that firms in our survey expect more efficiency gains from using AI via replacement of old capital than, from automating the workforce. In fact, the evidence of AI use for capital efficiency is extensive: 70% of AI investment in telecoms goes into network, for better planning of network coverage, equipment maintenance prevention (Balmer et al., 2020).

^4^AI measurement in ICT usage surveys: a review (oecd-ilibrary.org), see Table 2, and note from Statistics Canada, Eurostat, and US Census Bureau.

^5^This is in contrast with eg Rammer et al. (2022) whose average sampled German firm operate with ~100 by 2018 while this study average firm has a pool of about 1,500 workers by 2017.

^6^Highlights of the survey questionnaire are available in the original McKinsey and Company study, see mgi-artificial-intelligence-discussion-paper.ashxGoogle (mckinsey.com).

^7^See also Georgieff and Milanez (2021).

^8^Knoblach et al. (2020) synthesize the evidence of substitution between labor and capital and conclude that σ < 1; there is however no study on specific AI stock. Here we simplify such that σ = 1.

^9^More generally, γ = τn – 1, and τ = 1 if innovation is exclusive and leads to a monopoly.

^10^The logic here is that profit sharing signals lower wage base rate that increases the product margin of the firm. This product margin is a fortiori interesting to capture for winners take all innovations against rivals.

^11^Coverage of technology is an extensive margin measure. We use this measure following Manyika et al. (2017) and Bughin et al. (2018) which conclude that the effectiveness of AI technology to compete against work is not exhaustive, as assumed in our framework. E.g., 20% of work tasks may likely be not influenced at all by current set of AI technologies analyzed in this research.

^12^See among others AI Adoption Skyrocketed Over the Last 18 Months (hbr.org), or The state of AI in 2022—and a half decade in review | McKinsey.

^13^The probability of robots to reduce jobs has been shown to be higher in developed countries (Plumwongrot and Pholphirul, 2023).

^14^Our framework can easily accommodate this effect, where for instance, (7) generalizes to α = 1 – ϕ^σ^, where the elasticity of substitution σ < 1; firm may strategically invest in new skills, lowering σ, if more sticky employment leads to higher average labor productivity,ρ.
