# Supplementary material for: Does artificial intelligence kill employment growth: the missing link of corporate AI posture
Source: Front Artif Intell. 2023 Nov 17;6:1239466. doi: 10.3389/frai.2023.1239466 (PMC10691467; doi:10.3389/frai.2023.1239466)
Supplement: Supplementary file 1 [file Data_Sheet_1.docx]

**Appendix 1 : Key survey statistics**

**Table A. 1 – Sample features**

| **Industry** | | **Share** | **Country** | **Share** |
| --- | --- | --- | --- | --- |
| Professional Services | 16,4% | | Global | 26,2% |
| High technology | 11,5% | | Japan | 10,1% |
| Retail sales | 8,8% | | European Union, (outside the first 4) | 10,1% |
| Construction | 8,6% | | Italy | 8,1% |
| Health systems and services | 8,1% | | Canada | 7,7% |
| Financial Services | 6,1% | | The United States | 7,5% |
| Media and entertainment | 5,9% | | The United Kingdom | 6,9% |
| Education | 5,8% | | Asia, New Zealand, Australia | 6,9% |
| CPG | 5,7% | | France | 6,3% |
| Telecommunications | 5,3% | | Germany | 5,8% |
| Transport and logistics | 5,1% | | North America | 4,6% |
| Automotive and assembly | 4,9% | |  |  |
| Travel and tourism | 4,2% | |  |  |
| Energy and resources | 3,6% | |  |  |

**Table A.2 - AI technology diffusion, by sector, 2017**

| Sector | Adopted all AI's 'in percentage | |
| --- | --- | --- |
| High technology | 10,6 |  |
| Automotive and assembly | 10,4 |  |
| Construction | 3,4 |  |
| CPG | 8,5 |  |
| Retail sales | 4,1 |  |
| Media and entertainment | 1,9 |  |
| Telecommunications | 6,8 |  |
| Travel and tourism | 1,7 |  |
| Transport and logistics | 6,7 |  |
| Financial Services | 6,1 |  |
| Professional Services | 3,7 |  |
| Education | 3,8 |  |
| Health Care | 3,6 |  |
| Energy and resources | 5,1 |  |

| **Table A.3.: Summary statistics of the econometric variables** | | |
| --- | --- | --- |
| Variables | average | standard deviation |
| I | -9% | 48% |
| COVERAGE | 33% | 42% |
| DIFFUSION | 16% | 23% |
| NLEFFICIENCY | 69% | 57% |
| LEFFICIENCY | 24% | 16% |
| MARKETEXTENSION | 27% | 17% |
| MARKETSHARE | 26% | 21% |
| MARKUP | 1,15 | 32% |

**Appendix 2: IV results**

**Table A.4.: First-step regression**

|  | COVERAGE | DIFFUSION |
| --- | --- | --- |
| Current adoption in industries outside the one of focal firm | 0.43*** | 0,55*** |
|  | (0.045) | (0.071) |
| Capabilities: Stock of digital tech (wrt industry norm) | 0.13*** | 0.04*** |
|  | (0.003) | (0.002) |
| Focal firm is global | 0.093*** | 0.023 |
|  | (0.021) | (0.014) |
| Number of employees of focal firm | 0.04*** | 0.05* |
|  | (0.01) | (0.02) |
| Constant | -0.06 | -0.11 |
|  | (0.07) | (0.07) |
| Adjusted R² | 0.24 | 0.35 |
| Country dummies | Y | Y |
| Industry dummies | Y | Y |

Robust standard errors in parentheses.

F value for AI instruments in ADOPTION= 27,8***, in DIFFUSION= 35,2***.

* p<0,1; ** p<0,05; *** p<0,01

**Table A.5.: Probit estimates, job reallocation**

|  | Base line |  | IV |  |
| --- | --- | --- | --- | --- |
| COVERAGE | 0,181 | (6,12%)*** | 0,157 | (5,97%)*** |
| DIFFUSION | 0,185 | (6,98%)** | 0,154 | (8,66%)* |
| NLEFFICIENCY | - 0,009 | (-0,48%)* | 0,001 | (0,73%) |
| LEFFICIENCY | 0,155 | (7,98%)** | 0,160 | (8,16%)* |
| MARKETEXTENSION | - 0,011 | (-1,71%) | 0,003 | (0,21%) |
| MARKETSHARE | 0,071 | (1,88%)*** | 0,091 | (4,57%)** |
| MARKUP | 0,121 | (8,88%) | 0,061 | (4,93%) |
| Cst | 0,051 | (1,29)*** | 0,033 | (1,07)*** |
| Pseudo R² | 0,337 |  | 0,365 |  |
| Country and industry dummies | Y |  | Y |  |
| Cross-effects not included; sample restricted to no employment effects  Robust standard errors in parentheses. | | | | |
| * p<0,1; ** p<0,05; *** p<0,01. IV:Instrumental variable for ADOPTION and DIFFUSION as per thecomplete sample. | | | | |
|  | | | | |
